# A Case of an Ossifying Fibroma of the Mandible Suspected as a Static Bone Cavity

**DOI:** 10.7759/cureus.66104

**Published:** 2024-08-03

**Authors:** Noriyuki Sugino, Hiroko Kuroiwa, Katsumitsu Shimada, Takumi Sato, Akira Taguchi

**Affiliations:** 1 Department of Oral and Maxillofacial Radiology, Matsumoto Dental University, Shiojiri, JPN; 2 Department of Pediatric Dentistry, Matsumoto Dental University, Shiojiri, JPN; 3 Department of Clinical Pathophysiology, Matsumoto Dental University, Shiojiri, JPN; 4 Department of Oral and Maxillofacial Surgery, Matsumoto Dental University, Shiojiri, JPN

**Keywords:** inferior cortex of the mandible, mandibular canal, fibro-osseous lesion, benign tumor, static bone cavity, ossifying fibroma

## Abstract

Ossifying fibroma (OF) is a benign fibro-osseous lesion characterized by the proliferation of fibrous connective tissue containing immature bone and/or cementum-like hard tissue. Although the pathogenesis of OF remains unclear, trauma, previous extractions, and periodontitis are considered potential trigger factors. OF is more common in women aged from the second to fourth decades. Clinically, OF is characterized by slow-growing and asymptomatic swelling, often observed incidentally on radiological examinations. OF occurs more frequently in the mandible, particularly above the mandibular canal. Herein, we present a rare case of OF in an 18-year-old man initially misdiagnosed as a static bone cavity. The lesion was first observed as a radiolucent finding below the left mandibular canal on a panoramic radiograph. Later, cone-beam computed tomography (CBCT) imaging revealed the presence of calcifications within the lesion. Additionally, CBCT confirmed the presence of the lesion within the lingual cortical bone, revealing lingual swelling and thinning of the outer cortex. Enucleation was successfully performed under general anesthesia without any postoperative complications. Histopathological examination confirmed the diagnosis of OF, revealing mineralized tissue and proliferating fibrous connective tissue. This case underscores the challenges in diagnosing OF, particularly when it is located below the mandibular canal, emphasizing the importance of thorough imaging and differential diagnosis to avoid misinterpretation as a static bone cavity.

## Introduction

Ossifying fibroma (OF) is a benign fibro-osseous lesion characterized by proliferating fibrous connective tissue containing immature bone and/or cementum-like hard tissue [1]. While the pathogenesis of OF is unclear, trauma, previous extractions, and periodontitis are considered trigger factors [2]. OF tends to be more common in women, with a male-to-female ratio of 1:5, typically occurring from the second to fourth decades, although it can also occur in children, adolescents, and the elderly [1-3].

Clinically, OF presents as slow-growing and asymptomatic swelling, often observed incidentally on radiological examinations during dental checkups [1]. OF occurs predominantly in the mandible, particularly in the premolar-molar region, compared to the maxilla, but it can also occur in cranial and facial bones [1,2]. OF is usually a single lesion, though multiple lesions can occur in rare cases [1].

Typically, OF occurs above the mandibular canal in the cancellous bone of the mandible [3,4]. Herein, we present a rare case of OF in an 18-year-old man. It occurred within the inferior cortex below the mandibular canal and was initially diagnosed as a static bone cavity.

## Case presentation

**Figure 1:**
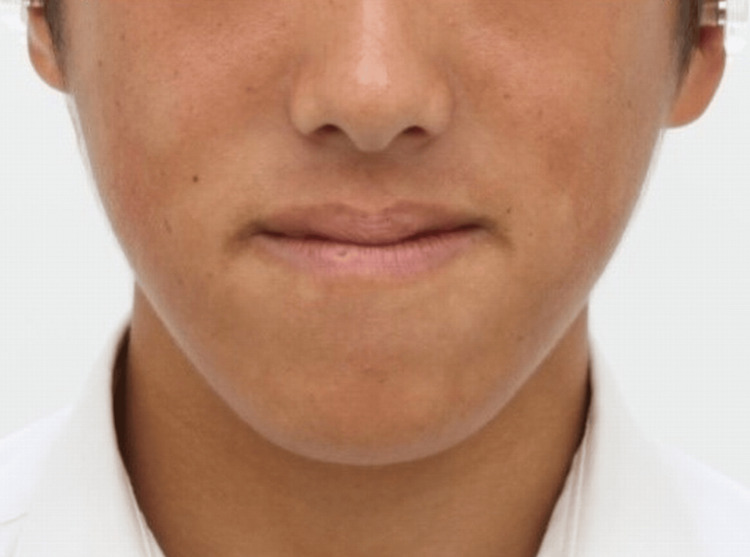
Facial photograph The patient exhibited a symmetrical face, with no observable abnormalities such as swelling or pain

An 18-year-old man presented to our dental hospital for orthodontic treatment. The patient had no significant medical history or family history and was asymptomatic with no issues in daily life. He exhibited a symmetrical face with no observable abnormalities such as swelling or pain (Figure 1). An intraoral clinical examination revealed no abnormalities, including mobilization of tooth, percussion pain, or paresthesia in the left mandibular molar region.

**Figure 2:**
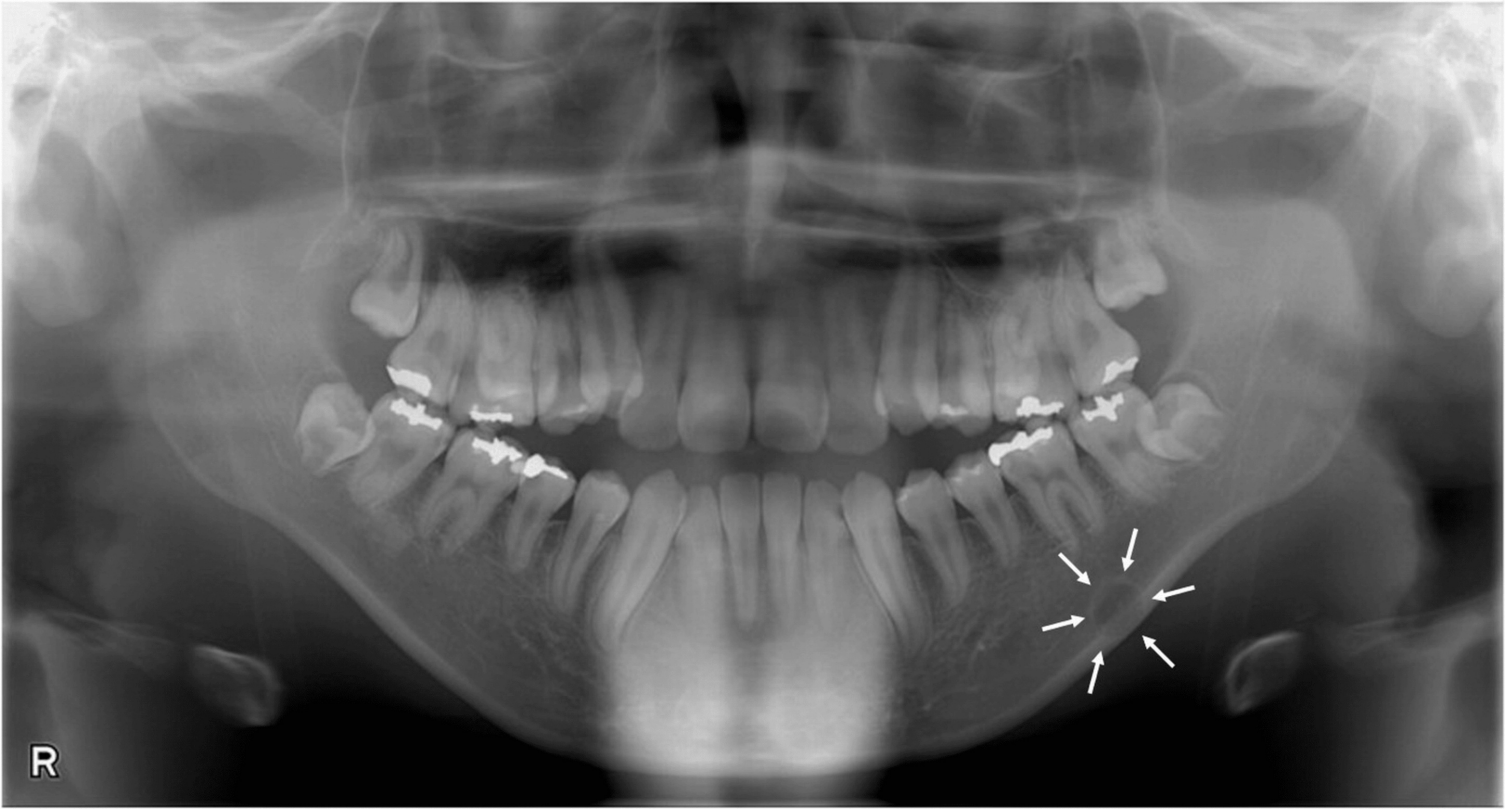
Panoramic radiograph A radiolucent lesion (surrounded by white arrows) is identified below the mandibular canal in the region of the left mandibular first molar

At the initial visit, a panoramic radiograph revealed a radiolucent finding below the mandibular canal in the region of the left mandibular first molar. The lesion, approximately 11×8 mm in size, had a well-defined margin, was circular in shape, was internally homogeneous, and had a unilocular appearance, with the lower part overlapping the mandibular cortical bone (Figure 2). Based on these radiographic characteristics, a static bone cavity was considered as a diagnosis. This lesion is typically characterized by its location below the mandibular canal, a well-defined margin, a circular shape, and a unilocular, radiolucent appearance. Additionally, the homogeneous internal structure and the absence of symptoms further supported this initial diagnosis. Due to these factors, the patient was kept under observation.

**Figure 3:**
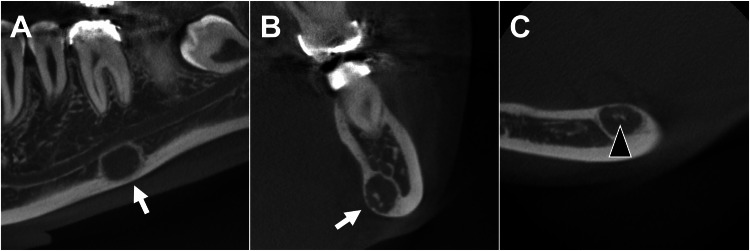
CBCT images The lesion (white arrows) is located below the mandibular canal (A: sagittal) and within the lingual cortical bone, showing swelling and thinning of the lingual cortex (B: coronal). A high-density area (black arrowhead) indicative of calcification is observed within the lesion (C: axial) CBCT: cone-beam computed tomography

Eight months later, cone-beam computed tomography (CBCT) images taken for orthodontic purposes incidentally revealed a radiolucent lesion in the same region. The lesion was located in the lingual cortical bone, showing swelling and thinning of the lingual cortex, with a high-density area within indicative of calcification (Figure 3). No displacement of the mandibular canal due to the lesion was observed.

As the lesion was within the planned osteotomy site, enucleation was performed concurrently with the osteotomy. The patient was monitored for approximately 14 months prior to surgery, during which no rapid growth or pain was observed in the lesion. Prior to surgery, a blood examination, including a complete blood count and biochemical assessments, was performed (Table 1). The results revealed elevated levels of white blood cells (WBC) and neutrophils (NEUTRO), while red blood cells (RBC), red cell distribution width (RDW), eosinophils (EOS), lymphocytes (LYMPH), total protein (TP), total cholesterol (T-cho), calcium (Ca), and iron (Fe) were found to be decreased. As planned, enucleation was performed simultaneously with the osteotomy under general anesthesia. The procedure was completed without complications, and the postoperative prognosis was favorable.

**Table 1:** Blood examination results WBC: white blood cell; RBC: red blood cell; HGB: hemoglobin; HCT: hematocrit; MCV: mean corpuscular volume; MCH: mean corpuscular hemoglobin; MCHC: mean corpuscular hemoglobin concentration; RDW: red cell distribution width; PLT: platelet; NEUTRO: neutrophils; EOS: eosinophils; BASO: basophils; MONO: monocytes; LYMPH: lymphocytes; TP: total protein; A/G: albumin/globulin; Alb: albumin; BUN: blood urea nitrogen; T-cho: total cholesterol; TG: triglycerides; Na: sodium; K: potassium; Cl: chloride; Ca: calcium; IP: inorganic phosphorus; Fe: iron; ALP: alkaline phosphatase; AST: aspartate aminotransferase; GOT: glutamic oxaloacetic transaminase; ALT: alanine aminotransferase; GPT: glutamic pyruvic transaminase; ChE: cholinesterase; CK: creatine kinase; γ-GT: gamma-glutamyl transferase; LDH: lactate dehydrogenase; T-Bil: total bilirubin

Parameter	Obtained value	Reference range
WBC count	17.5	4.0-11.0×10³ cells/µL
RBC count	4.61	4.7-6.1×10⁶ cells/µL (male)
HGB	14.0	13.8-17.2 g/dL (male)
HCT	41.9	40.7-50.3% (male)
MCV	90.9	80-100 fL
MCH	30.4	27-33 pg
MCHC	33.4	32-36 g/dL
RDW	11.8	11.5-14.5%
PLT count	231	150-400×10³ cells/µL
NEUTRO	86.5	40-70%
EOS	0.0	1-4%
BASO	0.1	0.5-1%
MONO	7.2	2-8%
LYMPH	6.2	20-40%
TP	6.3	6.4-8.3 g/dL
A/G ratio	1.86	1.0-2.5
Alb	4.1	3.5-5.0 g/dL
BUN	11.9	7-20 mg/dL
Creatinine	0.79	0.74-1.35 mg/dL (male)
T-cho	134	<200 mg/dL
TG	60	<150 mg/dL
Na	139	135-145 mmol/L
K	4.1	3.5-5.1 mmol/L
Cl	99	98-107 mmol/L
Ca	8.9	8.6-10.2 mg/dL
IP	4.1	2.5-4.5 mg/dL
Fe	20	65-176 µg/dL (male)
ALP	173	44-147 U/L
AST/GOT	17	10-40 U/L
ALT/GPT	19	10-40 U/L (male)
ChE	245	190-465 U/L
CK	152	52-336 U/L (male)
γ-GT	15	7-50 U/L (male)
LDH	176	140-280 U/L
T-Bil	0.57	0.1-1.2 mg/dL

**Figure 4:**
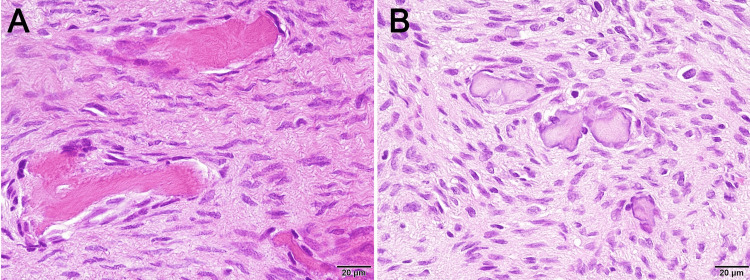
Histopathological images Histological examination reveals that the lesion consists of proliferating fibrous connective tissue, eosinophilic bony trabeculae with osteoblastic rimming (A), and psammomatoid bodies (B) (40× magnification)

Histopathological examination revealed that the lesion consisted of mineralized tissue and proliferating fibrous connective tissue (Figure 4). The mineralized tissue exhibited a mixture of trabecular and psammomatoid variants. The fibrous connective tissue was characterized by spindle cells and collagen fibers, proliferating in complex bundles, without evidence of distinct pleomorphism of the spindle cells. Based on these findings, the lesion was diagnosed as OF.

## Discussion

The World Health Organization (WHO) classification of OF has undergone several revisions. According to the latest classification in 2022, OF is defined as a benign mesenchymal odontogenic tumor and categorized into three variants: cemento-ossifying fibroma, juvenile trabecular ossifying fibroma, and psammomatoid ossifying fibroma [5].

The clinical manifestations of OF vary depending on the stage of lesion growth, with early lesions often being asymptomatic [6]. As the tumor progresses, it may lead to gradual swelling, significant functional impairment, facial asymmetry, displacement of the affected tooth, and root resorption. Clinical features such as pain and paresthesia are uncommon in OF [2]. In this case, there were no signs of facial asymmetry, swelling, tenderness, or paresthesia. The lesion was incidentally discovered during an X-ray examination for orthodontic treatment.

Panoramic radiographs, CBCT, computed tomography (CT), and magnetic resonance imaging (MRI) are essential tools for diagnosing OF [2]. Panoramic radiographs serve as the primary diagnostic modality, while CBCT and CT provide detailed insights into the internal structure of lesions. These imaging techniques are crucial for staging and accurately estimating the extent of tumors before surgery. Studies suggest that CT is either equivalent or superior to MRI in diagnosing OF [7]. MRI complements CBCT and CT by providing additional diagnostic accuracy when combined.

Panoramic radiographs typically reveal well-defined margins in OF, which are mostly unilocular, with approximately 20% presenting as multilocular lesions [8]. Lesions exhibit varying radiographic appearances depending on their stage, ranging from radiolucent to a mixture of radiolucent-radiopaque and eventually radiopaque as they mature [6]. As lesions enlarge, they may cause swelling of the jaw, thinning or perforation of the buccal and lingual cortical bone, and displacement of teeth. Tooth root resorption can occur; however, bone destruction is typically absent. CBCT and CT images reveal well-defined margins and internal variations based on the stage of the lesion. These modalities are superior to panoramic radiographs in assessing cortical bone changes and lesion extent. MR images generally show isointensity in fibrous tissue and hypointensity in osseous regions on T1-weighted images and hypointensity in both tissues on T2-weighted images [9]. In cases with immature fibrous tissue or fluid, T1-weighted images may appear hypointense, and T2-weighted images may appear hyperintense.

This case was initially diagnosed as a static bone cavity based on the panoramic radiograph, which observed a well-defined margin and unilocular radiolucency located below the mandibular canal, in contact with the inferior border of the mandible. According to the report by Collins et al. [3], while OF occurs most frequently in the mandible, cases that develop below the mandibular canal are extremely rare, accounting for only approximately 5.6%. Upon careful observation of the image, a punctate radiopacity can be seen slightly above the medial side of the cortical bone of the mandible within the lesion. However, diagnosing a punctate radiopacity as calcification is very difficult, and many dental radiologists are likely to interpret it as the trabeculae of cancellous bone. Furthermore, CBCT revealed that the lesion was located within the lingual aspect of the inferior cortex of the mandible. Typically, OF occurs in the cancellous bone and causes thinning or destruction of the cortical bone [4]. In this case, the disease developed within the cortical bone, causing it to appear split and expanded buccolingually. Such a case could not be found in previous reports. Due to these factors, it is presumed that the lesion was diagnosed as a static bone cavity at the initial visit. Therefore, as radiolucent lesions below the mandibular canal, such as in this case, may be misinterpreted as static bone cavities on panoramic radiographs and left untreated, it is essential to conduct thorough follow-up observations.

Histopathological examination is essential in determining preoperative and postoperative treatment plans [2]. The typical histopathological image of OF is characterized by proliferating fibrous connective tissue and immature bone and/or cementum-like hard tissue with a clear margin separating it from the surrounding existing bone [6]. The amount of hard tissue varies depending on the maturity of the lesion, and osteoblastic rimming is observed around it [10]. In our case, the typical histopathological features of OF were also observed, with the hard tissue exhibiting small trabecular and psammomatoid variants mixed with proliferating fibrous connective tissue.

The diseases that need to be differentiated from OF vary depending on the stage. In the early stages, OF resembles apical periodontitis, early cemento-osseous dysplasia, ameloblastoma, and central giant cell granuloma [11]. The mixed findings distinguish it from fibrous dysplasia, intermediate cemento-osseous dysplasia, condensing osteitis, calcifying epithelial odontogenic tumor, and adenomatoid odontogenic tumor [6,11]. In mature lesions, it is necessary to consider odontoma, late-stage cemento-osseous dysplasia, osteoblastoma, and osteosarcoma [6,11]. This case was diagnosed as a static bone cavity based on a panoramic radiograph. Subsequent CBCT suggested a calcified odontogenic cyst, a calcifying epithelial odontogenic tumor, and an adenomatoid odontogenic tumor. However, since the calcification within the lesion was minimal and there were no impacted teeth present, we believe that further diagnosis using radiological examination was limited.

The primary treatment modality for OF is surgical intervention [2,6]. Radiotherapy is contraindicated due to its potential to increase the risk of malignant transformation, estimated between 0.4% and 44% [2]. In this case, the lesion was located within the region that was enucleated during the osteotomy. The recurrence rate of OF varies in the literature, with MacDonald-Jankowski [8] reporting 12% and Adham and Dewi [12] reporting 14.4%. Furthermore, it has been reported that the recurrence rate differs by about 25% between cases with well-defined margins and those with indistinct margins on radiological findings [13]. Although the recurrence period is unpredictable, it ranges from six months to seven years after surgery, and annual follow-up is recommended for up to 10 years [2,10]. In this case, there has been no postoperative recurrence, and the patient's progress has been favorable.

## Conclusions

This case report highlights the diagnostic challenges in identifying OF. An 18-year-old male initially presented with a radiolucent lesion below the mandibular canal on a panoramic radiograph, suspected as a static bone cavity. Subsequent CBCT revealed internal calcifications within the lingual cortex of the mandible, and histopathological examination confirmed OF. The key finding is that radiolucent lesions below the mandibular canal on panoramic radiographs can be mistaken for static bone cavities, potentially leading to misdiagnosis and delayed treatment. This underscores the importance of thorough follow-up observations and detailed imaging, such as CBCT and CT, to ensure accurate diagnosis and appropriate management of such lesions.
